# Ultra-short pulsed laser welded-and-cut glass support pillars for vacuum insulating glass

**DOI:** 10.1007/s40940-025-00295-2

**Published:** 2025-04-28

**Authors:** Tara van Abeelen, Laura-Marie Mueller, Isabell Ayvaz, Franz Paschke, Adrian Dzipalski, Richard M. Carter, M. J. Daniel Esser, Gregor Schwind, Matthias Seel, Duncan P. Hand

**Affiliations:** 1 Applied Optics and Photonics Group (AOP), Institute of Photonics and Quantum Sciences, Heriot Watt University, Edinburgh, UK; 2 Glass Competence Centre, Institute of Structural Mechanics and Design, Technical University of Darmstadt, Darmstadt, Germany

**Keywords:** Laser welding, Laser cutting, Vacuum insulating glass, VIG, Glass spacer, Glass pillar

## Abstract

Vacuum insulating glass (VIG) has demonstrated competitive Ug-values (heat transfer coefficients) which show promise to further reduce energy consumption from buildings. Support pillars are an essential part of the design as they support the glass panes which would otherwise deform, and potentially collapse, under the pressure differential between the internal vacuum and the external atmosphere, however they act as small thermal bridges which contribute to heat transfer through the panes. The main cause for this is their high thermal conductivity as they are made out of metal. The use of glass support pillars would improve the Ug-value by 10–20% depending on the pillar size and pillar separation. Additionally, a directly bonded glass pillar, made from the same material as the glass panes, without the need for any adhesives, would improve recycling and visual appearance. We demonstrate a new technique for manufacturing glass support pillars using laser welding to bond, and laser cutting to shape the pillar to the substrate glass. We show that these pillars are able to withstand the expected atmospheric compressive force related to a pillar separation of 20 mm with promise for future research.

## Introduction

To achieve the European Union’s goal of climate neutrality by 2050, all sectors must be addressed. According to the International Energy Agency (Camarasa 2023), 30% of the world’s final energy consumption is used in buildings and the need to save energy in the building sector has led to tighter requirements for the insulation of buildings. A significant reduction in energy consumption in buildings can be achieved through the use of insulating glass units (IGU) in new buildings and in the retrofitting of existing buildings. International and national legislation, such as the UK Future buildings standard (2021), therefore sets a mandatory maximum value of 1.6 W/(m^2^K) for the heat transfer coefficient (Ug-value) that windows in new buildings must meet. Typical double glazed windows (argon filled with low-emission coatings) have Ug-values of 1.8 W/(m^2^K) (Cuce and Cuce 2016); with modern Vacuum Insulating Glass (VIG) achieving Ug-values of 0.4 W/(m^2^K), it is a promising and suitable product to support further energy reduction in building operations.

**Fig. 1:**
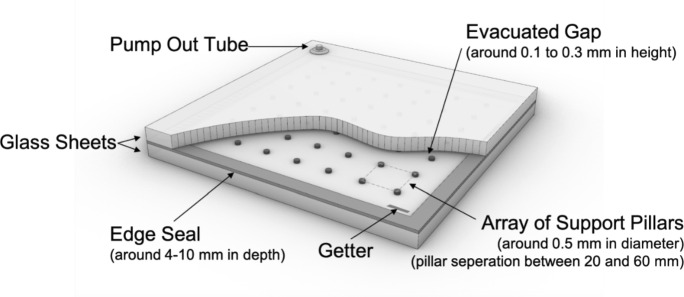
Typical setup of a vacuum insulating glass unit (not to scale)

The slim construction of a VIG and its high insulating properties are ideal for reducing energy loss through a building’s façade. Compared to modern triple-glazed insulating units (IGUs), a VIG can be a quarter of the glass thickness with the same or better Ug-value. This offers a high potential for material reduction, as not only the number of glass panes required can be reduced, but also the thickness of the window frame cross-section. At the same time, the handling of thinner and lighter window profiles on site is another advantage of single VIGs compared to triple IGUs (Fig. 1).

The standard single VIG consists of two glass panes hermetically sealed along the edge. The vacuum cavity is located between the two panes and is sealed by the edge seal. A vacuum of less than 0.1 Pa is generated in a variety of techniques depending on the production methods. In the “pump-out tube” method, the cavity is evacuated during the production process by means of a tube placed on one of the glass sheets (Collins and Simko 1998). Conversely, the “vacuum chamber” method does not require a pump-out tube as the VIG is evacuated and sealed in a single operation inside a vacuum chamber (Zhao et al. 2007). To keep the pressure low and constant during the operation of a VIG, a getter (reactive material) is used to bind to free molecules in the cavity (Collins and Simko 1998). Before a vacuum is created in the cavity, an array of pillars must be placed on the glass plate surface facing the cavity. These pillars are responsible for ensuring that the VIG withstands the stress caused by the pressure difference between the internal cavity and the external atmosphere and that the two glass plates do not deform towards each other. The pillars are essential in the construction of the VIG however, they also act as small thermal bridges and are therefore responsible for, among other things, increasing the heat transfer coefficient of the VIG. Standard pillars are made of high-strength steel materials, polymers, or ceramics. The methods used to position the pillars during the manufacturing process are complex, specialised, and proprietary to each manufacturer. Therefore, an alternative manufacturing method is desirable.

Research has been conducted on the design and material choice of the support pillars for reduced heat transfer between the pillars and the glass pane. It has been shown that the shape and size of the support pillar does in fact have a significant effect on the heat transfer (Zhang et al. 2022; Zhu et al. 2022), however, the thermal conductance of the material chosen is of more significance (Collins and Simko 1998; Zhu et al. 2020). The design of the pillar array in the vacuum cavity can be critical in adjusting the thermal transmittance of the VIG. The thermal conductivity of the pillar array is dependent on the spacing, size and material properties of the pillars, and can be calculated according to ISO 19916-1 (2018). Using a standard low-emission (low-E) coating (ε = 0.02), which minimises the radiative heat transfer coefficient in the vacuum cavity, the heat transfer coefficient of the pillar array accounts for 75–95% of the total heat transfer coefficient of the VIG unit, depending on the pillar spacing. Standard pillars, made out of metal, possess a high thermal conductivity and additionally reduce the transmission of light and cause a visual obstruction due to their opacity. Glass has a low thermal conductivity and is transparent so is a desirable alternative. Furthermore, it simplifies recycling if the pillar is produced from the same material as the glass panes.

The use of glass as the support pillar material has been investigated in the literature. One technique is screen printing with sintering of glass pastes (Zhao et al. 2013) which, although providing good uniformity in size, is accompanied by long drying times and is a multi-step process. Another technique explored uses laser irradiation to create dome-like features directly in the substrate (Streltsov et al. 2014; Kocer 2017). Whilst showing promise for using laser sources in VIG manufacturing, restrictions for the feature sizes possibly apply.

In this manuscript, proof of concept of a novel technique for manufacturing the support pillars using ultra-short pulsed (picosecond-femtosecond) laser welding and cutting is presented. Laser welding is used to directly bond the thin glass to the substrate, whilst laser cutting provides accurate shaping of the pillar. The advantage of using these techniques includes high processing speeds (Soref 2010), high repeatability, and uniformity in size. Laser welding is an established technique for the direct bonding of glass-glass (Cvecek et al. 2019). Through tightly focusing a laser beam, with high intensity ultra-short pulses, at the interface between the two glass samples, nonlinear absorption occurs which leads to the heating and subsequent melting of the glass. The cooling and fusing of this molten glass can bridge a small gap and directly bonds the two parts (Morawska et al. 2024). Femtosecond pulsed ablation-based laser cutting can be used to cut the thin glass (Markauskas et al. 2023) used for the pillar in a clean and precise way, providing the necessary small circular features. Laser welding and cutting of glass, as established techniques, have been demonstrated extensively in literature (Nordin et al. 2016; Wlodarczyk et al. 2019; Markauskas et al. 2023; Chhadeh et al. 2025), including for soda lime glass, a standard material in VIGs (Crimella et al. 2023; Jia et al. 2023; Huang et al. 2025) however, these techniques have not, to the authors’ knowledge, been explored in combination for VIG pillar manufacturing, despite the clear potential.

## Proof of concept

**Fig. 2:**
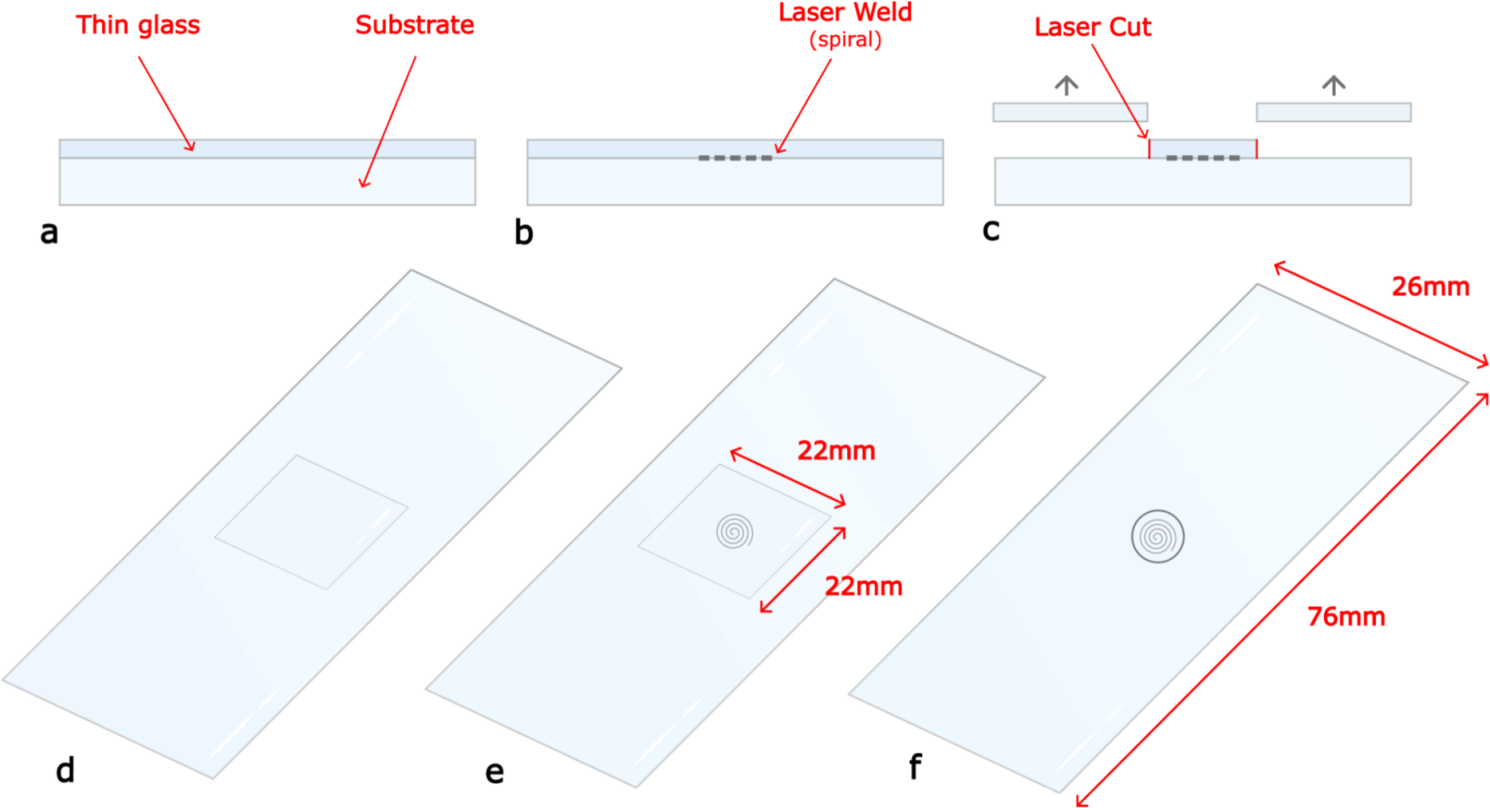
Schematic of the glass pillar manufacturing process. **a** The thin glass and microscope slide are placed in optical contact after a thorough cleaning procedure. **b** A 1 mm outer diameter spiral weld bonds the thin glass to the microscope slide. **c** A 1.2 mm diameter circle is cut out of the thin glass, around the weld, which shapes the pillar. **d** corresponding perspective view of **a**, **e** corresponding perspective view of **b**, **f** corresponding perspective view of **c**

To demonstrate this proof of concept, we laser welded a thin glass coverslip to a glass substrate and laser cut through the coverslip leaving a circular support pillar directly bonded to the substrate, with the pillar height determined by the thickness of the coverslip. This is shown in Fig. 2. The material chosen was soda lime glass for both the coverslip and substrate, in accordance with material standards for VIGs (2008; 2018). Initial work was caried out using standard microscope slides of thickness 1.1 mm (± 0.1 mm) to simulate the window glass substrate, with further testing conducted on 50 mm diameter optical windows of thickness 3.00 mm ± 0.20 mm to better represent windowpanes with their larger thickness The coverslip thicknesses are in the range 130–190 μm, which is comparable to standard pillar heights for VIGs.

Research has been conducted on the optimum size of the support pillars with Zhang reporting that a radius of >0.2 mm meets the minimum strength requirements (Zhang et al. 2022). Collins reiterates that there is no exact correct dimension for the pillars, but the material and hence thermal conductivity is of most importance (Collins and Simko 1998). In our work, the focused laser spot is translated through a 1 mm outer diameter Archimedean spiral to form the weld, with inner diameter of 0.6 mm and pitch of 0.2 mm. This weld pattern was chosen to follow the shape of the circular pillar, and to cover most of its surface area to ensure a secure bond to the substrate. A 1.2 mm circular cut is then used to separate the coverslip material around the weld, allowing for any error that occurs between locating the welding and cutting clamps as well as the linear stage movement which forms the pillar. Both these sizes were chosen arbitrarily for this proof of concept study.

To assess the potential of glass pillars in VIGs, parameter studies were carried out on the thermal conductivity of the VIGs (Ug-value). The calculation of the Ug-value was performed using the analytical formulas outlined in the ISO 19916-1 standard (2018). For a standard VIG with one low-E coating (emissivity of ε = 0.02), one quarter of the thermal transmittance through the centre of the VIG is due to radiative heat transfer and the other three quarters are due to direct heat transfer through the pillar array. Therefore, the size and material of the pillars are critical in manipulating the Ug-value of the VIG.

Table 1 shows a comparison of the Ug-value for metal (Stainless-Steel with thermal conductivity λ_p_ = 15 W/(mK)) and glass (thermal conductivity λ_g_ = 1 W/(mK)) pillars for a VIG as a function of pillar radius, r_p_, for different pillar separations, l_p_, and a pillar height, d_p_, of 0.2 mm. A low-E coating set at ε = 0.02 was used for all calculations. For our chosen pillar radius of 0.6 mm, a minimum separation of 40 mm is required to achieve the state-of-the-art Ug-value for VIGs (0.3 to 0.7 W/(m^2^K)). The load, force, on each pillar is calculated using the pillar separation and atmospheric pressure, taken as 0.1 MPa.

**Table 1:** The load on each pillar to due to atmospheric pressure from the vacuum for different pillar separations

Pillar separation [mm]	20	30	40	50
Ug—value of metal pillar [W/m^2^K]				
r_p_ = 0.6 mm	2.11	1.21	0.79	0.56
r_p_ = 0.5 mm	1.88	1.06	0.68	0.49
r_p_ = 0.25 mm	1.34	0.62	0.41	0.30
Ug—value of glass pillar [W/m^2^K]				
r_p_ = 0.6 mm	1.89	1.06	0.69	0.49
r_p_ = 0.5 mm	1.63	0.90	0.58	0.42
r_p_ = 0.25 mm	0.86	0.47	0.32	0.24
Load on one pillar [N]	40	90	160	250

This data shows that using glass instead of metal for the pillars can reduce the Ug-value of the VIG by 10–20% depending on the pillar radius and the pillar separation. To further reduce the Ug-value in VIGs, the pillar radius can be reduced.

## Experimental arrangement

**Fig. 3:**
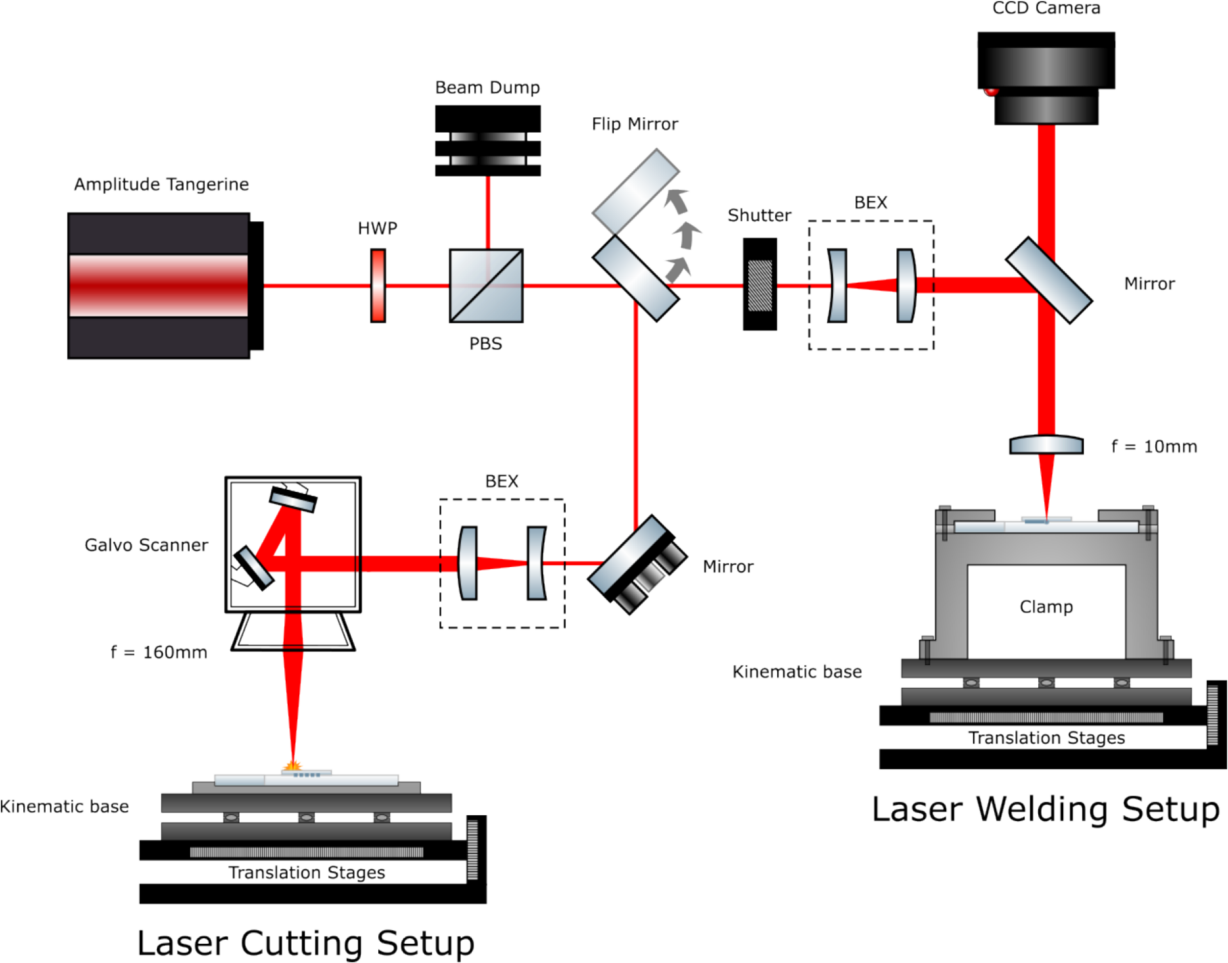
Experimental arrangement for the laser welding and cutting of the glass support pillars. HWP—Half waveplate, PBS—Polarising beam splitter cube, BEX—Beam expanding telescope. The flip mirror allows for redirecting the laser between the welding and cutting setups

The experimental arrangement is shown in Fig. 3. This includes the Amplitude Tangerine laser (1030 nm, 260 fs-10 ps, >35 W, >2 MHz) and a set of motorised stages. These stages provide X, Y movement (Aerotech ANT95), with a resolution of ± 0.5 μm. A half waveplate (HWP) and a polarising beam splitter cube (PBS) are used to controllably attenuate the power. The flip mirror switches the laser beam between the welding and cutting setups. Both beam expanding telescopes (BEX) increase the gaussian beam size to a 1/e^2^ diameter of 8.9 mm, whilst an external shutter (Thorlabs SH05) allows the beam power to be synchronised with the stages during processing. For the welding setup, a fixed 10 mm focal length objective (Thorlabs AL1210M-B) is used, and the sample is placed in a clamping jig and onto the motorised stages secured by a locking magnetic mount (Thorlabs KBM1/M). A CCD camera was used to find the top surface of the coverslip, through calibrated measurement of the surface reflections of the laser from the glass. For the cutting setup, a galvanometer scanner (LS-Scan XY) is used with a 167 mm focal length objective (F-Theta-Ronar 4401-531-000-26).

## Laser-based manufacturing

### Laser welding

#### Methodology

Laser welding directly bonds the thin glass to the microscope slide. The laser focus must be set at the interface to ensure a successful bond and avoid in-bulk modifications. This position was found using the CCD camera by identifying surface reflections from the coverslip and translating the focus through the coverslip to the interface, calibrated for the materials refractive index, and a spherical surface aberration model. This model is based on geometric calculations (Huot et al. 2007) of both longitudinal and transverse aberrations due to the varying amounts of refraction for each of the constituent rays of the laser beam due to focusing light below the surface of a transparent material of refractive index >1 (in this case soda-lime glass). By calculating both the longitudinal and transverse aberrations for different focusing depths a minimum transverse aberration, that corresponds to the smallest spot size (focus) can be found for the relative longitudinal aberration, and hence located at the interface. Through this method, the thickness of each sample can be independently considered and ensures the focus can be placed at the interface regardless of sample thickness. The calibration between the camera and the laser focus position was found by scanning multiple lines of the same parameter at different z-positions and observing their position relative to the interface through cross sectional analysis. A value of 150 μm was found and is applied to all the samples alongside their individual depths of focus associated with their thickness.

Although not explicitly required for laser welding it is desirable for the sample to be in optical contact prior to welding to aid in bridging the interface effectively (Chen et al. 2015; Jia et al. 2023). This was achieved through a thorough cleaning procedure and careful mounting of the glass samples in a clamping jig. Cleaning consisted of wiping the microscope slides with acetone using Integrity Polyester/Cellulose wipes until clean to visual inspection. Each slide was then individually wrapped in a wipe and submerged in a 1:10 mixture of Neutracon, a cleaning agent, to deionised water (DI) water before entering the ultrasonic bath for 15 min at 30 degrees. Parts were then rinsed with DI water whilst still wrapped before being rewrapped and immersed in only DI water for a second ultrasonic bath of the same settings. The microscope slides were then dried with Nitrogen gas and wiped with acetone again. At the same time the coverslips were also wiped with acetone, these are unsuitable for the same procedure due to their delicate nature. Once both are wiped, they are pressed into contact by applying an even pressure by hand. Optical contact is established through observing interference fringes (Newton’s rings), where the fringes correspond to the (very small) gap between the sample, the aim being to have regions with no fringes. Although non-contact laser welding has been demonstrated (Chen et al. 2015; Jia et al. 2023), and may be more preferrable in industrial applications, the authors opted for optical contact laser welding to minimize potential sources of errors for this proof of principle. Once optical contact is established, the sample is placed in the clamping jig which applies a consistent force on the sample by means of a torque screwdriver set to 0.8 Nm, equivalent to 1 kN, which applies a total estimated force of 3 kN to the sample due to a three-screw arrangement. This force was chosen through visual observation of the interference fringes and provided enough force without breaking the glass samples. This allowed for repeatability between samples. The jig is designed to hold each sample in the same position each time, allowing for spatial repeatability. This is achieved using an alignment disk that sits in the jig. The disk is a thin aluminium sheet with a cutout matching the corresponding sample size, allowing the sample to sit inside the cutout and remain centred relative to the jig. The force applied supports the optical contact. Clamping was not required for the cutting process as the sample is already bonded, instead only the alignment disk was used for spatial repeatability.

**Fig. 4:**
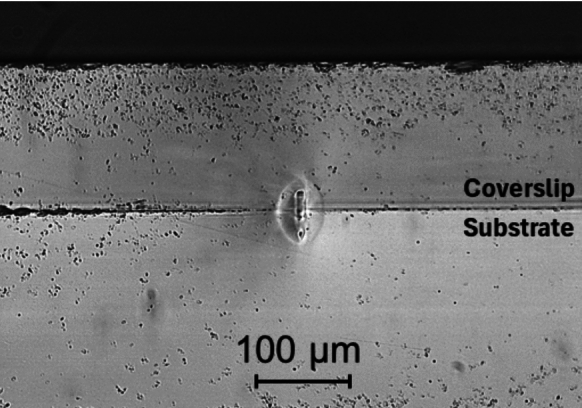
Cross section microscope image showing the found optimum Z-position. As shown, this results in the weld located across the interface

Figure 4 is an image showing the final z-position offset used. The central feature is where plasma occurred and formed the weld, whilst the surrounding area is the resolidified melt zone.

#### Results

**Fig. 5:**
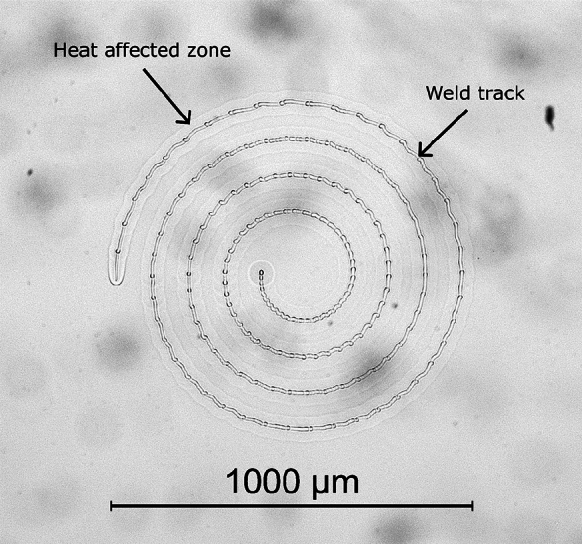
Bright-field (BF) microscope image of the weld

There are several parameters involved in the laser welding process that can be optimised, such as pulse length, pulse repetition rate, average power, translation speed, focusing conditions and clamping (Morawska et al. 2024). A pulse duration of 5 ps and a repetition rate of 500 kHz was chosen based on our previous laser welding work (Wlodarczyk et al. 2019; Morawska et al. 2024). The average power was optimised through a parameter map, with ~0.5–1.0 W found to produce acceptable welds. A power of 0.68 W (hence a pulse energy of 1.36 μJ) was chosen as it falls within this range and corresponds to one of the discrete power increments available on the laser system. Significantly higher powers were observed to cause cracking in the glass whilst lower powers were insufficient to cause material modification and hence did not form a bond. An optical microscope was used to analyse cracking, whilst the bond strength was investigated by using a scalpel to prise the coverslip off the substrate; if the coverslip removes easily, an insufficient bond was made suggesting more power is required. The scan pattern chosen is a 1 mm outer diameter spiral translating from the outside to in, at a programmed speed of 2 mm/s, with a typical weld shown in Fig. 5. The spiral approach enables a larger area to be welded to replicate a spot weld whilst avoiding the need for parameter compensation in the vicinity of tight turns or corners which may cause cracking due to creation of stress regions. More importantly a spiral is a continuous motion therefore is more suited than, for example, a series of concentric circles.

By examining the weld spiral in Fig. 5, the region of glass that has been melted and re-solidified (the heat-affected zone) can be seen as highlighted, creating a good join along the full length of the spiral. Dark field imaging showed no evidence of cracking (not included here as it is uniformly dark in the absence of any cracks). There is a slight wobble to the weld path which is due to the motorised stage calibration and does not affect the process. To determine the success of the weld, attempts to remove the coverslip from the microscope slide were performed, using a scalpel, which resulted in the coverslip breaking around the outer edge of the weld track, indicative of a successful bond. As previously mentioned, cross sectional analysis of samples was carried out to find the z-position, which also confirmed successful welding. Cross sectional analysis also enables the feature size to be measured, as seen in Fig. 4; in our case, this was roughly 50 μm in height. Since the feature is relatively long, it provides some vertical tolerance, allowing for slight adjustments in positioning, making it more suited for industrial applications, where tolerance is required due to potential errors in machinery. The feature size can be increased through, for example, increasing average power (Wlodarczyk et al. 2019).

The welding process was easily translated to the thicker 3 mm samples due to the ease of finding focus with the help of the CCD camera and the aberration model.

### Laser cutting

#### Methodology

The aim of the cutting process is to cut a circle in the coverslip around the spiral weld, without inducing damage in the substrate. Such damage is likely to affect the strength of the glass which in our application could lead to failure of the glass pane at the pillar site. Laser cutting enables the excess coverslip to be removed, leaving a cylindrical pillar of a chosen diameter. The cutting is completed after the welding process because if the processes were reversed, the clamping, optical contact procedure, and the positioning of a small glass pillar becomes extremely complex.

Here, we utilise top-down ablation-based femtosecond pulsed laser cutting which is a well-established technique producing reliable and repeatable cutting results in glass at high processing speeds due to using a Galvanometer optical scanner. The femtosecond (rather than picosecond) pulse duration is advantageous for cutting due to its reduced thermal accumulation effects as the pulse duration is shorter than the time for thermal diffusion to occur in the material (Markauskas et al. 2023). This allows for increased quality of cuts.

#### Results

The first attempt was carried out on coverslips and microscope slides in optical contact but not yet welded, with the aim of ensuring the coverslip is fully cut through but also easily removeable from the microscope slide to observe damage. A successful weld would prevent the ability to remove the coverslip.

A pulse duration of 260 fs was used. Other parameters were optimised to reduce the processing time as much as possible, a key consideration for industrial relevance. The cutting parameters used in the final demonstration includes 50 repetitions at 50 mm/s at 13.53 W and a repetition frequency of 10 kHz. For each spacer, the cutting process took approximately 33 seconds. Further optimisation would likely reduce this time significantly. The combination of the welding and cutting process adds a source of error as, in our implementation, the sample needs to be removed from the welding clamp and re-positioned for cutting. As discussed above, to ensure that the weld is fully contained inside the cut, the pillar diameter was chosen to be 1.2 mm diameter. However, it should be noted that some of the welds were slightly off-centre within the cut pillar, which could be improved by using better jigging.

**Fig. 6:**
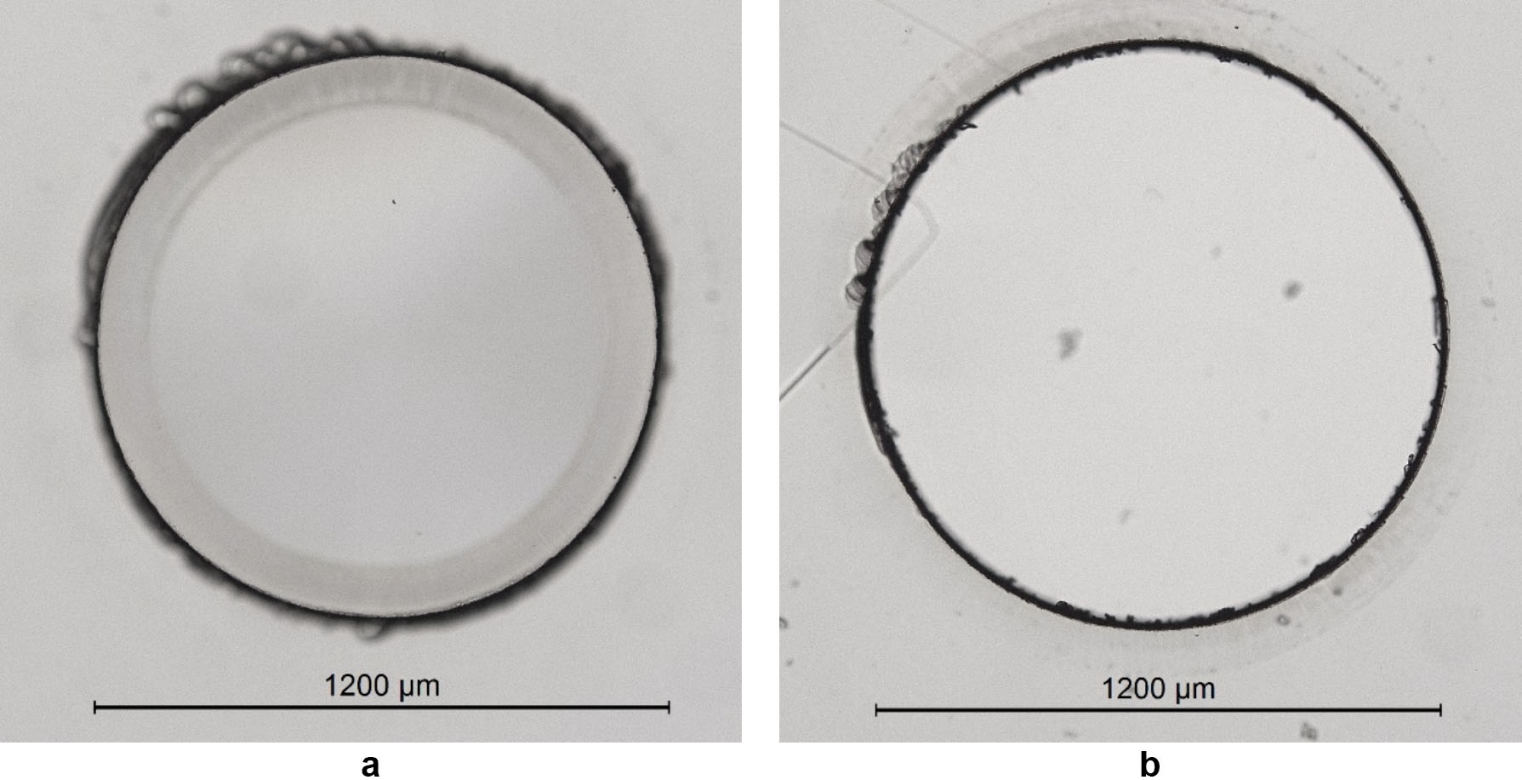
**a** the pillar post laser cutting, **b** the removed coverslip

**Fig. 7:**
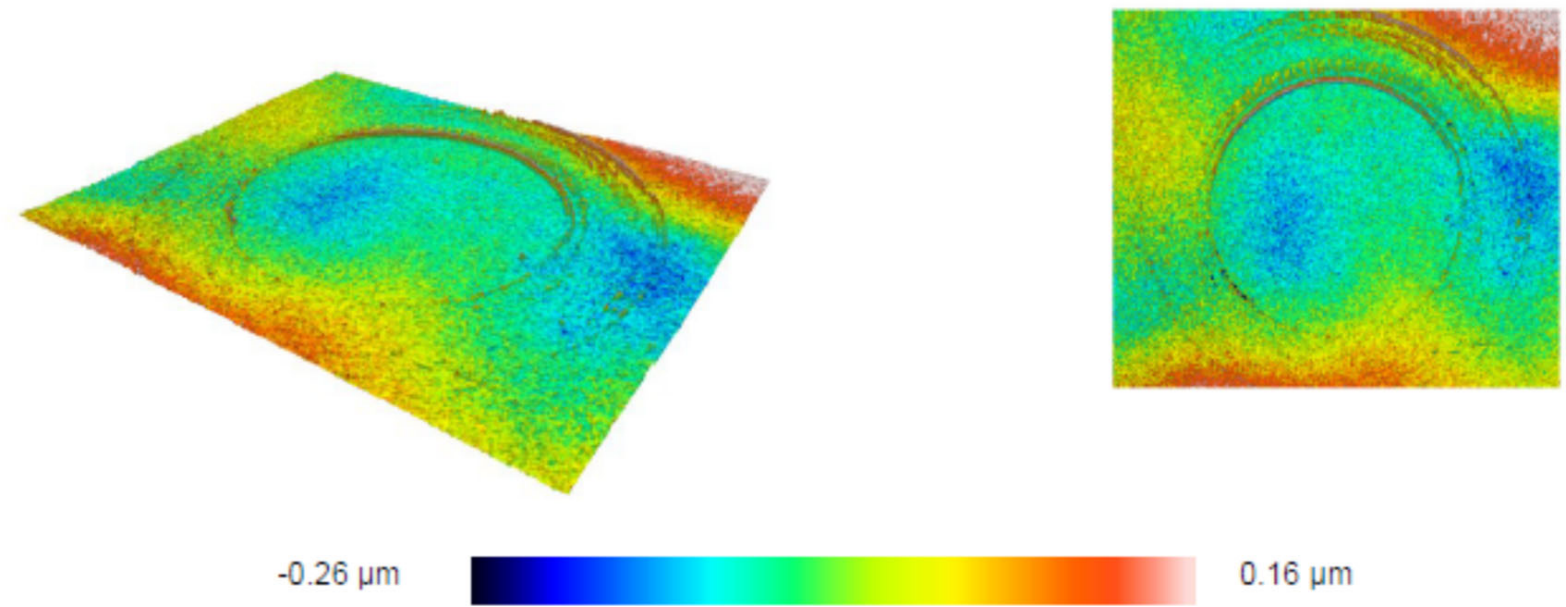
Surface profile of deposited debris for a sample in optical contact. The volume of deposited material is 1.711e-5 mm^3^. The surface profile confirms the addition of material rather than the removal. The resultant surface roughness is equal to 7.7 nm

Figure 6 shows the results of the cutting process to produce a 1.2 mm diameter pillar. The black ring is observed under the microscope due to the cutting process resulting in a bevelled edge. As observed in Fig. 6a there are ‘scalloped’ edges at the base of the pillar which suggests that the glass was not fully cut through before the excess material was broken away, leaving the intact pillar. This was purposely done to avoid damaging the microscope slide however the pillar is nevertheless easy to extract from the surrounding coverslip. Once removed we inspected the microscope slide and damage was observed to be minimal, however there are signs of debris deposited onto the slide surface from the coverslip. Contact imperfections allows the debris from the cutting process to be spread across the surface of the microscope slide away from the line of the cut. The deposited debris is minimal as the coverslip was in optical contact before cutting and not observable by eye. This was confirmed to be deposited debris through measuring the surface profile with a Sensofar (S neox) and should not contribute to damage in the microscope slide nor affect the strength (Fig. 7).

**Fig. 8:**
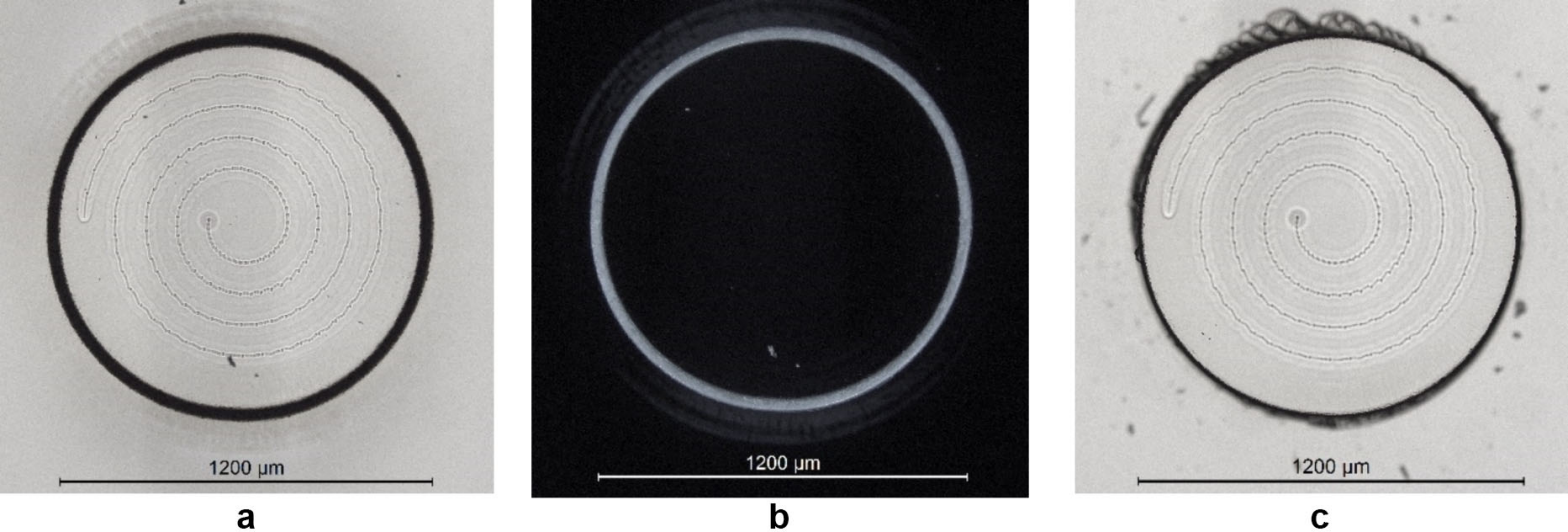
Microscope images of the final support pillar. **a** Bright field image of the weld and cut prior to removing the coverslip, **b** corresponding dark field image of **a**, **c** Bright field image of the support pillar with excess coverslip removed

The cutting and separation process was found to be highly repeatable between samples. As shown in Fig. 8a the cut was confined to the beam path, with no cracking as shown in Fig. 8b. Figure 8c shows the sample once the excess coverslip was broken off and removed, leaving a clean edge to the pillar. For this application it is most important that the cut edge close to the top surface is cut smooth, as this will be in contact with the other windowpane and would therefore cause a point of stress. One significant thing to reiterate is that each coverslip and microscope slide vary in thickness. For our work we did not relocate the focus or adjust the number of repetitions according to the thicknesses for cutting. This should be explored in future work to optimise the process.

## Mechanical testing and evaluation

### Indentation test

Due to the evacuation of air from the VIG, the pillars must permanently withstand atmospheric pressure, equivalent to 0.1 MPa, leading to a high compression force. For a rectangular array with 20 mm spacing, a pillar has to withstand a permanent force of 40 N, as previously indicated in Table 1. To determine the feasibility performance of these glass pillars, indentation testing was conducted. These tests were completed on the microscope slide samples.

**Fig. 9:**
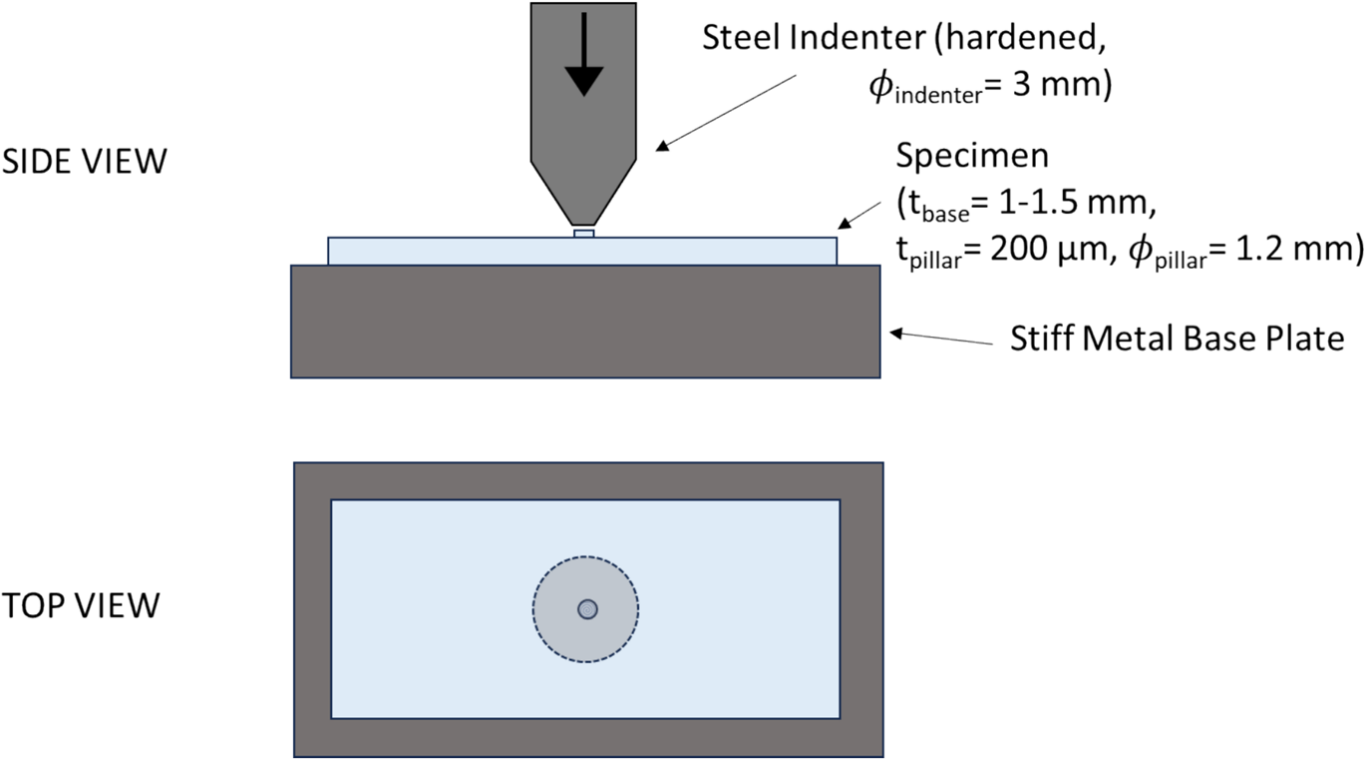
Schematic side and top view of the indentation test arrangement. These tests were conducted on the microscope slides

A schematic view of the experimental arrangement is provided in Fig. 9. The test setup and execution were realised and carried out based on the European Assessment Document 300021-00-0404—Annex C.1. For the test, a universal testing machine (ZWICK ROELL Z05) was used to apply compression force. The samples used were welded and cut as previously discussed. For this testing batch, the series ‘K’ consisted of 8 samples. The samples were positioned on a stiff metal base to eliminate any deformation of the microscope slide during testing. A load was applied using a cylindrical hardened metal (high-strength steel) indenter, of diameter 3 mm, and was increased using a path control of 1 mm/min. The load applied was stepped through 3 valued with each sample, 50 N, 100 N and 250 N for a proof of principle test. After each loading and unloading, a microscope image was taken to visually inspect for any damage, such as cracks. As a final step the load was increased until ultimate failure was reached.

**Fig. 10:**
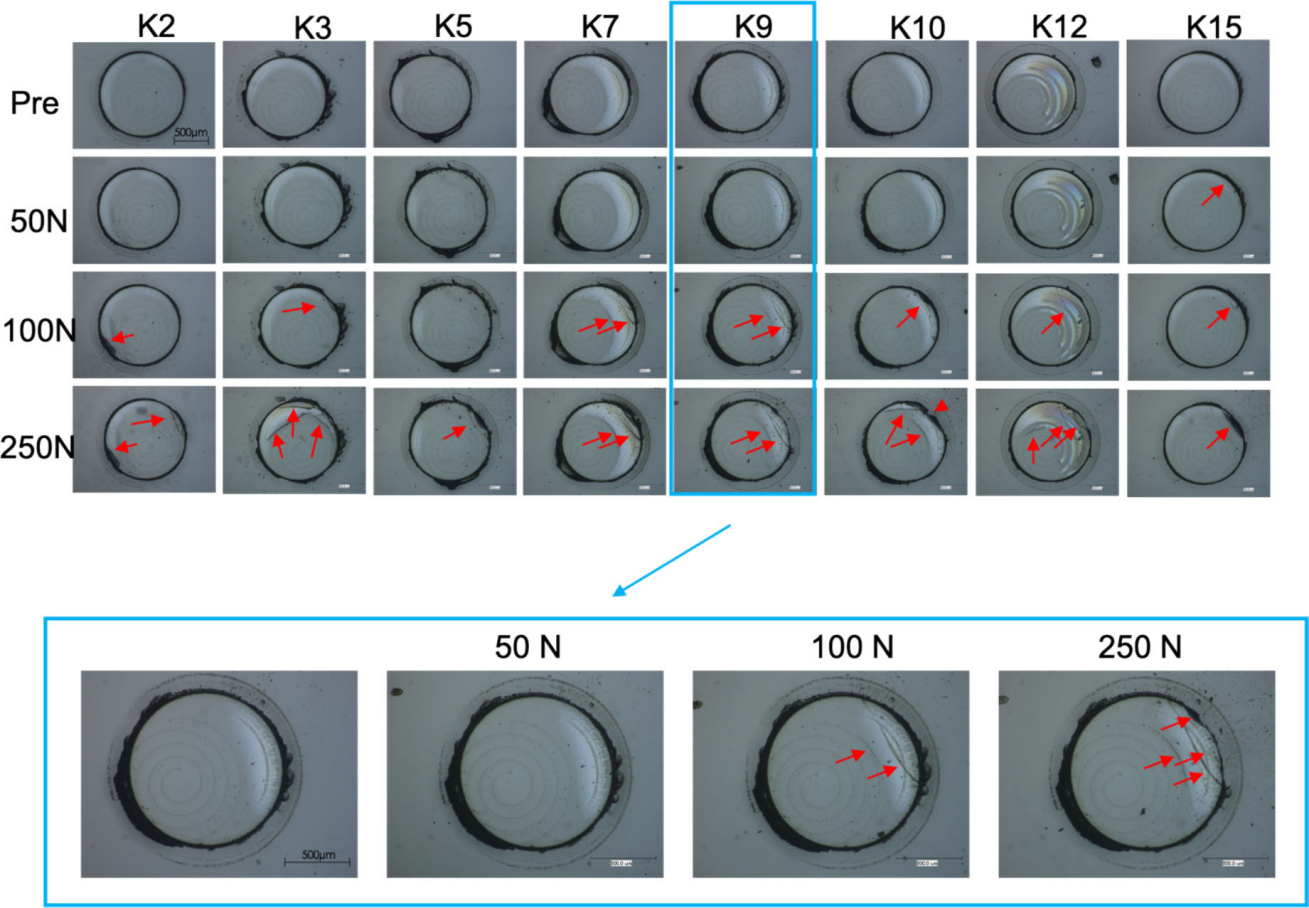
Pillar region before and after indentation testing. The red arrows highlight the areas where damage was visually detected

Figure 10 shows the microscope images of the tested samples at each stage. These pictures were taken using a Keyence Light Microscope (VHX 600) using coaxial light. Due to the experimental setup, continuous tracking of the cracks was not possible, hence incremental loading and unloading was performed. The exact point, and hence load, at which cracks occurred is therefore not determined. Typically, radial cracks occur during loading and lateral cracks occur during the unloading phase. The cracks inspected in these tests are assumed to be radial. After the 50 N load was applied, there was no observed damage in all but one sample (K15). Small cracks occurred after the 100 N load, typically forming at the edge of the pillar penetrating into the pillar but not into the underling glass panel. As previously mentioned, in some cases the weld and cut were not perfectly centred relative to each other due to clamping. For these samples, the cracks were observed at the edge furthest from the weld, at a cantilever position. This could be avoided through improved clamping and positioning of sample. Additionally reducing the cut diameter to match the weld diameter would remove this discrepancy. When further increasing the load to 250 N existing cracks grew or new cracks were formed, again at the edge of the pillar. When increasing the load further with the aim of determining the point of ultimate failure of the pillar, the base plate was instead damaged and was thus not further investigated. If additional tests are performed, thicker base plates would be advantageous. It is worth mentioning that a potential discrepancy in parallelism between the indenter and the pillar may have introduced additional stress concentrations, which could have contributed to the observed cracks. However, this cannot be stated definitively and would require further investigation. It is important to note that, despite the crack formation, due to the weld, the pillars remained intact even under loads up to 250 N. This demonstrates the potential for these welded glass pillars in VIGs.

### Coaxial bending test

**Fig. 11:**
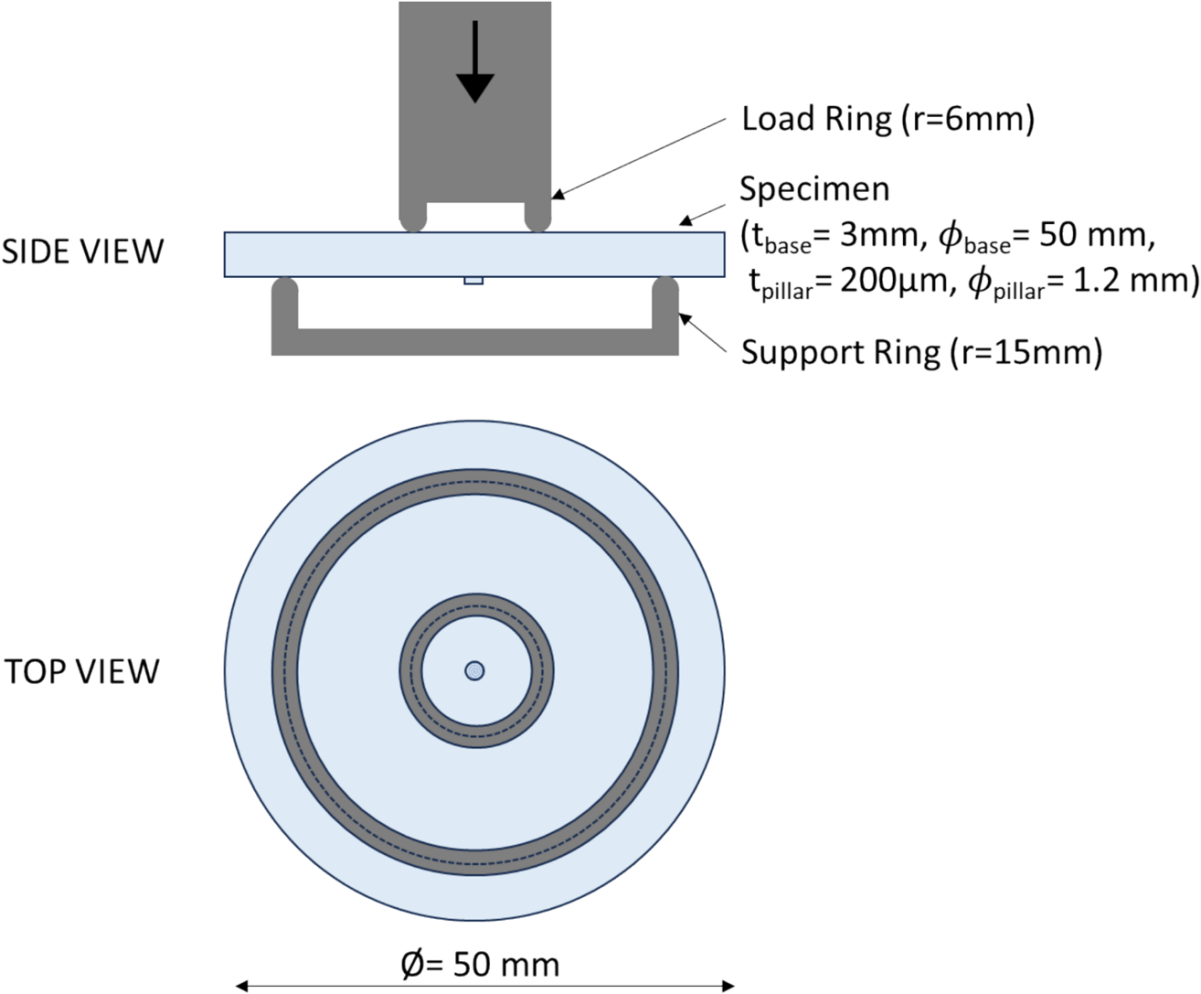
Schematic side and top view of the coaxial bending test

To investigate whether the welding and cutting processes influence the strength of the substrate, coaxial double ring bend tests were performed using a setup adapted from the principles outlined of EN 1288-5 (2000). Typically, a 6/30 ratio is used; however, a 6/15 ratio was applied here due to the sample size. The same 6/15 ratio setup was used for the reference set, unprocessed samples without pillars, ensuring that the results are directly comparable. These tests are typically used to determine the bending strength of glass samples in the construction industry and hence are also used in this ‘proof of concept’ study. A schematic overview of the test setup is provided in Fig. 11.

Each sample was on average 2.95 ± 0.1 mm thick and had a diameter of 50 ± 0.2 mm. A support ring with a diameter of 30 mm and a load ring of diameter 12 mm was chosen. For this testing batch, the series ‘B’ consisted of 10 samples. Samples were equipped with a thin sticky foil on the compression side to allow determination of the crack origin after testing.

For this test, a universal testing machine (ZWICK ROELL Z050) was used. The load was increased until failure occurred. A force corresponding to a stress rate of 2 ± 0.4 MPa/s, required by EN 1288-5 was applied using force control.

**Fig. 12:**
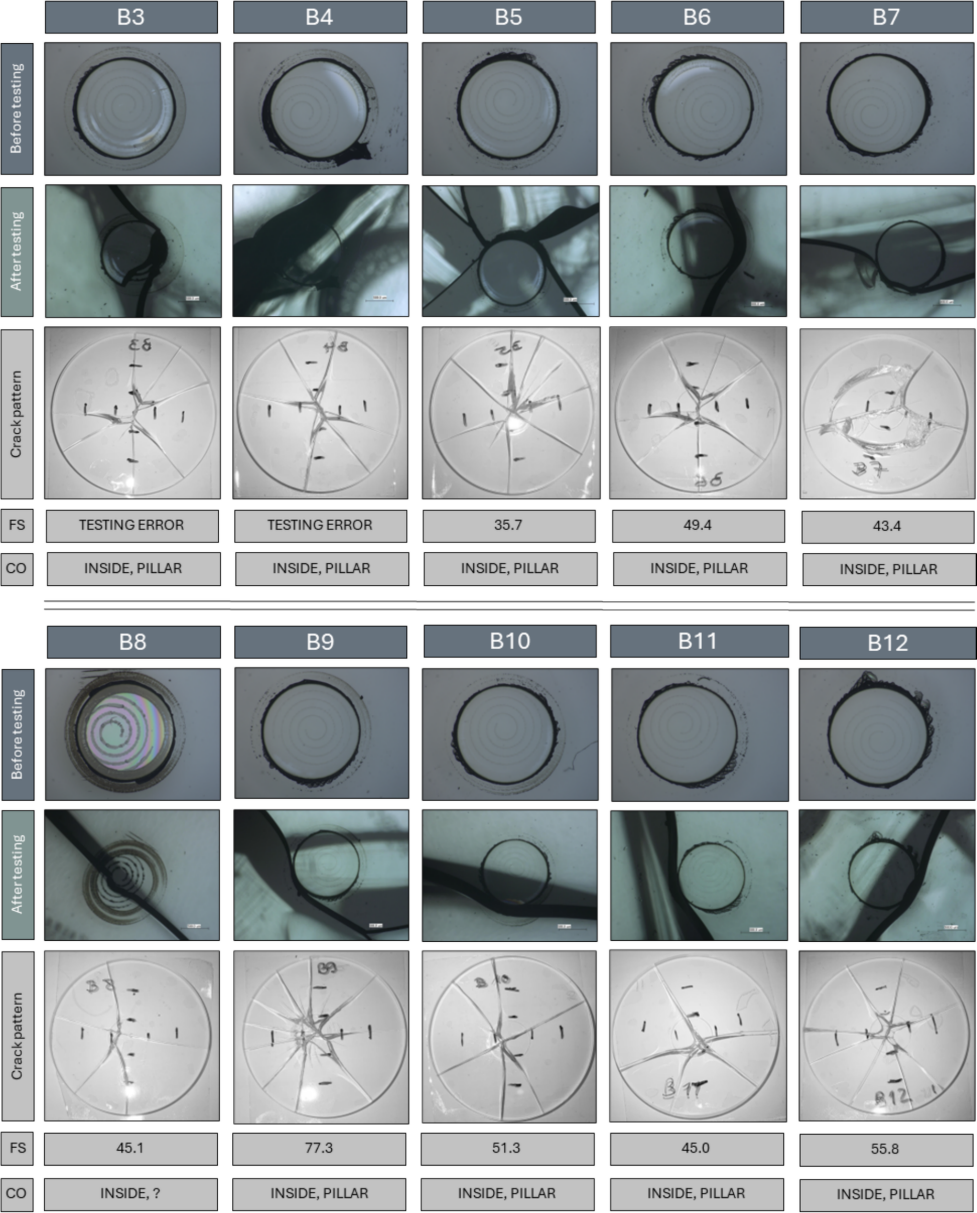
Specimen before and after coaxial bending testing. 1st row: Samples before testing, 2nd row: Samples after testing, 3rd row: crack pattern, 4th row: the stress at failure (‘FS’) and 5th row: the location of the crack origin (‘CO’). The ring diameter of rows 1 and 2 is 1.2 mm (pillar) whilst row 3 shows the whole sample of diameter of 50 mm

Microscopic images of the tested samples are shown in Fig. 12, including the area around the pillar before and after testing. These pictures were taken using a Keyence Light Microscope (VHX 600) using coaxial light. The crack pattern images were captured using a mobile microscopic device. The failure stress is determined using Eq. 1 out of EN 1288-1 (2000). Poisson’s ratio is set to 0.23 for soda-lime silicate glass.

**Fig. 13:**
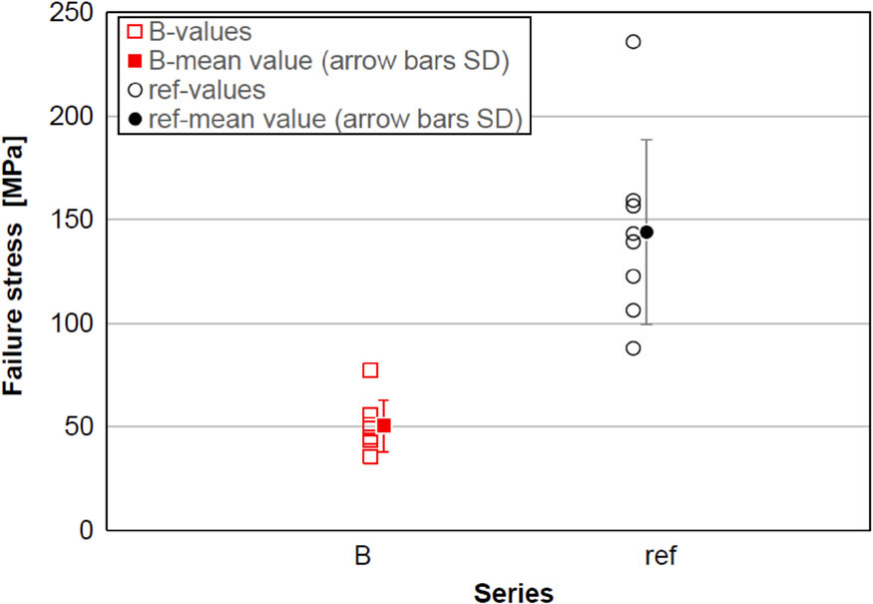
Results of coaxial double ring of series B and ref (single value, mean value with standard deviation (arrow bars))

For sample B3 and B4 force control issues caused the samples to be loaded at a rate much higher than the 2 MPa/s which was intended. Yet, in both cases the pillar region still seems to have led to failure. In Fig. 12, the position of the crack origin (‘CO’) is given. With one exception, the pillar has caused a weakness and influenced the propagation of the crack. As can be seen in Fig. 12, 2nd row, in all cases but sample B8, the crack travelled along the edge of a pillar, which leads to the assumption that welding or cutting induced either Eigenstresses or surface flaws near the pillar edge. In order to evaluate the potential strength reduction of the welded samples, coaxial double ring tests were carried out on a reference series with 10 samples. Series ‘B’ (welded) and the reference series were statistically analysed. The results are listed in Table 2. Figure 13 visualises the individual values and mean values with standard deviation (SD, single) for each series. The mean value of the fracture stress of series ‘B’ (welded pillars) is 50 MPa (standard deviation 12 MPa), whilst that of the reference series ‘ref’ is 144 MPa (standard deviation 12 MPa). A correlation test (Spearman) between the failure stresses and series (presence/absence of the pillar) indicates that there is a statistically significant difference between the two series (*p*-value = 1.3E-5), with a correlation value of 0.86, i.e. a strong correlation between the presence, or absence, of the pillar and the corresponding strength according to Cohen (Cohen 1988).

**Table 2:** Results of the coaxial bending test (series B and reference samples)

Overview	Unit|series	B	Ref
Total number of valid samples	[piece]	8/10	9/10
Fracture/crack origin	–	Inside load ring (7 × pillar)	Inside load ring
Failure stress—mean value	[MPa]	50	144
Failure stress—minimum	[MPa]	36	88
Failure stress—maximum	[MPa]	77	236
Failure stress—standard deviation (SD)	[MPa]	12	45
Failure stress—coefficient of variation (CoV)	–	0.25	0.31

Although it is not entirely clear which of the two processes, welding or cutting, lead to a reduction in the strength of the substrate, the authors believe that the welding process was likely responsible. This conclusion is made based on the initial laser cutting work, which showed that deposited debris was observed rather than material removal, indicating no damage to the substrate, as seen in Fig. 7. Additionally, we ensured that the cut didn’t fully penetrate the coverslip, to avoid damaging the substrate, evidenced by the ‘scalloped edge’ as observed in Figs. 6 and 8. The weld, however, is essentially a surface defect on the substrate and hence expected to slightly alter the materials response to the bending testing. The lower strength can result due to the change in the local geometry in relation to the “welding spot”. This change can lead to high local stresses in the area of the weld and thus to the lower strength in relation to the reference series. In principle, this can be checked by a finite analysis calculation of the local welding area. Whether this significantly impacts a real VIG glass pane is unclear as this proof of concept was demonstrated on a small scale. Additionally, standard glass panes would be larger in size but also consist of multiple pillars. Despite this, the impact of the weld could be minimised by reducing the volume of material that is melted during the welding process, for example through reducing the pillar diameter, the spiral pitch or altering the scan strategy to, for example, a singular circle rather than a spiral. Furthermore, the welding parameters could be optimised further, or this proof of concept could be investigated with thicker substrates (as our 3 mm thick sample is at the lower end of thicknesses typically used) or on toughened glass to better represent materials used in industry and are less likely to experience significant strength loss.

## Industrial relevance and future work

These findings show promise for the future of glass support pillars helping to improve the efficiency and appearance of VIG units. As previously mentioned in the manuscript, the laser weld feature allows for some vertical tolerance and the optical contacting of samples is not explicitly required making this process more suited for industry. Additionally, the methods developed could be integrated into production lines using automated systems, such as robotic arms, along with suitable monitoring to ensure quality.

Future work should focus on optimising the laser welding process to minimise its impact on the strength of the substrate, using techniques previously suggested. These include reducing the pillar diameter or pitch, altering the scan strategy to reduce the amount of material melting such as a singular circle rather than a spiral, and further optimising the parameters used. Furthermore, producing a small-scale prototype of a VIG with these pillars would be invaluable in fully assessing the feasibility and suitability of these glass pillars, their manufacturing technique, and to verify the performance of a VIG unit thereby validating the concepts presented in this study for practical industrial applications.

## Conclusions

The potential move from metal to glass support pillars in VIG would reduce the Ug-value and visual obstruction. It has been shown that the switch from metal to glass reduces the Ug-value by 10–20%. Here, we show that laser welding and cutting forms a potential manufacturing process to produce these glass pillars. The welding successfully provides direct bonding of the pillar material to the substrate without any cracking, while the laser cutting process enables the pillar to be cut to size accurately and repeatably.

Pillar size and separation directly relate to the force on each individual pillar due to the vacuum. Indentation tests were conducted and showed that pillars can withstand a compressive force of at least 50 N which is more than the force at a pillar due atmospheric pressure acting on a VIG with pillar array of 20 × 20 mm^2^. However, for these 1.2 mm diameter pillars, a separation of 40 mm or more is required to achieve state-of-the-art Ug-values which corresponds to a force of 160 N or higher. With higher loads, cracks were formed. These cracks could be mitigated with improved clamping, which would prevent a weld-cut position mismatch, or matching the cut diameter to the weld diameter, and hence eliminate the observed cantilever effect. However, despite crack formation, the pillars still remained in place due to the weld, even under loads of 250 N. Future improvements are suggested.

Coaxial double ring tests on the base of EN 1288-5 show that the welded and cut pillars (series B) reduce the strength by a factor of mean value 2.9 compared with a reference series without pillars. Since the weld is essentially a surface defect on the substrate, and due to the nature of this test, it is expected to show an effect on the strength of the substrate. To reduce the impact of the weld on the substrate strength is discussed with improvements such as reducing the pillar diameter or welding to toughened glass is recommended. For the design of future VIG units with these welded-on pillars, this must be considered. This work shows promise for further research.

## Data Availability

The data for Table 1 and Fig. 13/Table 2 have been uploaded with access link: 10.17861/375595c3-d39f-4d6a-9f7f-ddea0c98bacc.

## References

[CR1] (2000) EN 1288-1. Glass in building—Determination of the bending strength of glass - Part 1: Fundamentals of testing glass

[CR2] (2000) EN 1288-5. Glass in building—Determination of the bending strength of glass - Part 5: Coaxial double ring testing on flat specimens with small test surface areas

[CR3] (2008) ISO 16293-1 Glass in building—Basic soda lime silicate glass products - Part 1: Definitions and general physical and mechanical properties, pp 1–4

[CR4] (2018) ISO 19961-1 - Glass in building—Vacuum insulated glass. Part 1: Basic specification of products and evaluation methods for thermal and sound insulating performance

[CR5] (2021) The Future Buildings Standard. Consultation on changes to Part L (conservation of fuel and power) and Part F (ventilation) of the Building Regulations for non-domestic buildings and dwellings; and overheating in new residential buildings, pp. 39–40

[CR6] Camarasa, C.: The Energy Efficiency Policy Package: Key Catalyst for Building Decarbonisation and Climate Action. ArchDaily (2023)

[CR7] Chen, J., Carter, R.M., Thomson, R.R., Hand, D.P.: Avoiding the requirement for pre-existing optical contact during picosecond laser glass-to-glass welding. Optics Express **23**(14), 18645–18657 (2015) 26191923 10.1364/OE.23.018645

[CR8] Chhadeh, P.A., Sleiman, K., Hoffmann, H., Funke, N., Rettschlag, K., Jäschke, P., Baitinger, M., Knaack, U., Kaierle, S., Seel, M.: Design of additively manufactured glass components for glass point fixings. Glass Struct Eng **10**, 66 (2025)

[CR9] Cohen, J.: Statistical power analysis for the behavioural sciences. Lawrence Erlbaum Associates Publishers, New York (1988)

[CR10] Collins, R.E., Simko, T.M.: Current status of the science and technology of vacuum glazing. Solar Energy **62**(3), 189–213 (1998)

[CR11] Crimella, D., Jwad, T., Demir, A.G.: Microcutting of glass with high ablation efficiency by means of a high power ps-pulsed NIR laser. Optics Laser Technol **166**, 109645 (2023)

[CR12] Cuce, E., Cuce, P.M.: Vacuum glazing for highly insulating windows: recent developments and future prospects. Renew. Sustain. Energy Rev. **54**, 1345–1357 (2016)

[CR13] Cvecek, K., Dehmel, S., Miyamoto, I., Schmidt, M.: A review on glass welding by ultra-short laser pulses. Int. J. Extreme Manuf. **1**(4), 42001 (2019)

[CR14] Huang, Y., Zhang, Z., Wang, X., Li, L., Yang, L., Li, M., Zhu, W.: Process study of picosecond laser welding of soda-lime glass. Optics Laser Technol. **182**, 112095 (2025)

[CR15] Huot, N., Stoian, R., Mermillod-Blondin, A., Mauclair, C., Audouard, E.: Analysis of the effects of spherical aberration on ultrafast laser-induced refractive index variation in glass. Optics Express. **15**(19), 12395–12408 (2007) 19547610 10.1364/oe.15.012395

[CR16] Jia, X., Li, K., Li, Z., Wang, C., Chen, J., Cui, S.: Multi-scan picosecond laser welding of non-optical contact soda lime glass. Optics Laser Technol **161**, 109164 (2023)

[CR17] Kocer, C.: A Novel Glass Spacer for Vacuum Insulated Glazing. GPD Glass Performance Days, Tampere (2017)

[CR18] Markauskas, E., Zubauskas, L., Račiukaitis, G., Gečys, P.: Femtosecond laser cutting of 110–550 µm thickness borosilicate glass in ambient air and water. Micromachines (Basel) **14**(1), 176 (2023) 36677237 10.3390/mi14010176 PMC9867199

[CR19] Morawska, P., Dzipalski, A., Van-Abeelen, T., MacKay, P., Carter, R., Esser, M.J.D., Hand, D.: Industrial picosecond pulse laser welding of stainless-steel to quartz for optical applications. Opt. Mater. Express **14**(11), 2588–2599 (2024)

[CR20] Nordin, I.H.W., Okamoto, Y., Okada, A., Takekuni, T., Sakagawa, T.: Effect of focusing condition on molten area characteristics in micro-welding of borosilicate glass by picosecond pulsed laser. Appl. Phys. A Mater. Sci. Process **122**(5), 1–11 (2016)

[CR21] Soref, R.: Mid-infrared photonics in silicon and germanium. Nat. Photonics **4**(8), 495–497 (2010)

[CR22] Streltsov, A., Dickinson, J., Grzybowski, R., Harvey, D., Logunov, S., Ozturk, A., Sutherland, J., Potuzak, M.: Laser texturing of doped borosilicate glasses. SPIE - Laser Applications in Microelectronic and Optoelectronic Manufacturing XV 7584 (2014)

[CR23] Wlodarczyk, K.L., Hand, D.P., Maroto-Valer, M.M.: Maskless, rapid manufacturing of glass microfluidic devices using a picosecond pulsed laser. Sci. Rep. **9**(1), 20215–20213 (2019) 31882878 10.1038/s41598-019-56711-5 PMC6934552

[CR24] Zhang, Y., Yuan, W., Han, L., Zhang, R., Xi, X.: Multi-objective design for critical supporting parameters of vacuum-insulated glazing with a case study. Appl. Sci. **12**(15), 7504 (2022)

[CR25] Zhao, J., Luo, S., Zhang, X., Xu, W.: Preparation of a transparent supporting spacer array for vacuum glazing. Vacuum **93**, 60–64 (2013)

[CR26] Zhao, J.F., Eames, P.C., Hyde, T.J., Fang, Y., Wang, J.: A modified pump-out technique used for fabrication of low temperature metal sealed vacuum glazing. Solar Energy **81**(9), 1072–1077 (2007)

[CR27] Zhu, Q., Wu, W., Yang, Y., Han, Z., Bao, Y.: Finite element analysis of heat transfer performance of vacuum glazing with low-emittance coatings by using ANSYS. Energy Build. **206**, 109584 (2020)

[CR28] Zhu, W., Zhang, S., Shin, S., Gorti, S., Shah, B., Joshi, P., Bhandari, M.: Effects of pillar design on the thermal performance of vacuum-insulated glazing. Constr. Build. Mater. **316**, 125724 (2022)

